# First-line Aspiration Thrombectomy with the RED43 Catheter for Acute Ischemic Stroke due to Medium or Distal Vessel Occlusion

**DOI:** 10.1007/s00062-025-01567-8

**Published:** 2025-09-29

**Authors:** Vera Aebischer, Alex Brehm, Nikki Rommers, Alejandro Spiotta, Mohammad Sowlat, Karthik Raghuram, Justin M. Cappuzzo, Jeffrey Beecher, Charles Matouk, Abdelaziz Amllay, Axel Rohr, Manraj Heran, David Volders, Pascal J. Mosimann, Mohammad Al-Tibi, Johan Wassélius, Björn M. Hansen, Markus Holtmannspötter, Martin Schlösser, Stephan Meckel, Oumaima Aoua, Suresh Giragani, Andrea Boghi, Nikolaos Ntoulias, Victor Schulze-Zachau, Ioannis Tsogkas, Kristine A. Blackham, Aikaterini Anastasiou, Marios Psychogios

**Affiliations:** 1 Department of Diagnostic and Interventional Neuroradiology, University Hospital Basel, University of Basel, Petersgraben 4, 4031 Basel, Switzerland; 2 Department of Clinical Research, University of Basel, University Hospital Basel, 4031 Basel, Switzerland; 3 Department of Neurosurgery, Medical University of South Carolina, 29425 Charleston, SC USA; 4 Reno Radiological Associates and University of Nevada, Mill St., 1155 Reno, NV USA; 5 Endovascular and Cerebrovascular Neurosurgery, Atlantic Brain and Spine, 28412 Wilmington, NC USA; 6 Department of Neurosurgery, Yale University, 333 Cedar Street, 06510 New Haven, CT USA; 7 Vancouver General Hospital & UBC Hospital, Department of Radiology/Neuroradiology, University of British Columbia, Vancouver, British Columbia Canada; 8 Division of Neuroradiology, Joint Department of Medical Imaging, Toronto Western Hospital, University Health Network, Toronto, Ontario Canada; 9 Division of Neuroradiology, Joint Department of Medical Imaging, Toronto Western Hospital, University Health Network, University of Toronto, Toronto, Ontario Canada; 10 Stroke Imaging Research Group, Department of Clinical Sciences Lund, Lund University and Department of Radiology, Skåne University Hospital, Lund, Sweden; 11 Department for Diagnostic & Interventional Neuroradiology, Paracelsus Medical University, Breslauer Str. 201, 90471 Nuremberg, Germany; 12 RKH Kliniken Ludwigsburg, Institute of Diagnostic and Interventional Neuroradiology, Posilipostr. 4, 71640 Ludwigsburg, Germany; 13 Apollo Hospitals, Hyderabad, Telangana India; 14 S.G. Bosco Hospital, Neuroradiology, Torino, Italy

**Keywords:** Stroke, Endovascular thrombectomy, Medium or distal vessel occlusion, First-line technique, Aspiration

## Abstract

**Purpose:**

Techniques for endovascular thrombectomy in acute ischemic stroke due to medium or distal vessel occlusions (MDVO) need to be improved, and first-line aspiration is emerging as a promising technique. The objective was to describe first-pass and final reperfusion rates with a triaxial direct aspiration first pass technique (ADAPT) and a novel, quadriaxial technique (QUATTRO-ADAPT) using RED43 Reperfusion Catheters to target MDVOs.

**Methods:**

This retrospective study collected data from 11 stroke centers in Europe and North America between 2023 and 2025. Patients with primary, isolated MDVOs who underwent thrombectomy using first-line aspiration with RED43 Reperfusion Catheters were included. Primary outcome was first-pass successful reperfusion rate (modified Treatment In Cerebral Infarction score ≥ 2b). Additionally, final reperfusion rates, complications and functional outcomes up to 90 days were evaluated.

**Results:**

We included 85 cases. Median age was 74 years (IQR 67–81) and median National Institutes of Health Stroke Scale score 9 (IQR 6–15). The majority (69.4%) had an occlusion of the co- or nondominant M2 segment of the middle cerebral artery. First-pass successful reperfusion was 63.5% overall, with 55.6% for the triaxial ADAPT and 69.4% for the quadriaxial QUATTRO-ADAPT. Final successful reperfusion was achieved in 96.5%. Complications included vessel perforations (2.4%), subarachnoid hemorrhage (3.5%), parenchymal hematoma type 2 (2.4%), and symptomatic intracerebral hemorrhage (1.2%). Functional independence (modified Rankin Scale 0–2) at 90 days was observed in 58.7%.

**Conclusion:**

This study shows that first-line aspiration, especially in a quadriaxial approach, can be effective and safe. These findings should be evaluated in prospective trials.

**Supplementary Information:**

The online version of this article (10.1007/s00062-025-01567-8) contains supplementary material, which is available to authorized users.

## Introduction

Acute ischemic stroke substantially contributes to global mortality and morbidity, with 25–40% of cases attributable to medium or distal vessel occlusions (MDVO) [1, 2]. For large vessel occlusions, endovascular thrombectomy (EVT) has been shown to be a highly effective treatment [3] and a direct aspiration first-pass technique (ADAPT) to be an effective treatment alternative to using stent retrievers (SR) [4–6]. However, recent randomized controlled trials (EnDovascular therapy plus best medical treatment (BMT) versus BMT alone for MedIum VeSsel Occlusion sTroke—a prAgmatic, international, multicentre, randomized triaL (DISTAL) and EndovaSCular TreAtment to imProve outcomEs for Medium Vessel Occlusions (ESCAPE-MeVO Trial)) investigating the role of EVT in MDVO have not shown a benefit on functional outcomes, and underscore with successful reperfusion rates around 70–75% the need for improved EVT techniques [7, 8]. Which thrombectomy technique is most effective as a first-line approach for MDVO treatment remains a subject of ongoing debate.

The RED43 Reperfusion Catheter (RED43; Penumbra, Alameda, USA), a novel aspiration catheter designed to target MDVOs, has been available since April 2023 in the US, and May 2024 in Europe but data of EVT with RED43 in MDVO remain scarce. Assessing novel techniques enabled by such devices is crucial, as EVT in MDVO raises new challenges due to thinner vessel walls, increased tortuosity and small perforator arteries that can be avulsed. To address these features, this study evaluates the efficacy of a quadriaxial setup, consisting of a guide catheter, a larger aspiration catheter, a smaller aspiration catheter (such as the RED43), and a microcatheter and/or microwire. Using this setup for first-line aspiration will be referred to as “QUATTRO-ADAPT”.

This retrospective study describes first-pass and final successful reperfusion rates of first-line aspiration with RED43 in patients with acute ischemic stroke due to primary, isolated MDVOs. Procedural complications and functional outcomes are reported. Furthermore, we describe reperfusion rates of a quadriaxial first-line aspiration technique, QUATTRO-ADAPT.

## Methods

The study received approval from the applicable ethics committee (Project-ID 2025-00139) on February 24, 2025. Cases from April 2023 to January 2025 were collected from 11 stroke centers in Canada, Germany, Italy, Sweden, Switzerland and the US. Eligible cases included patients aged 18 years or older, had been diagnosed with acute ischemic stroke due to a primary, isolated MDVO, and were treated with first-line aspiration using a RED43. Patients with documented refusal for research consent were excluded. MDVO were defined according to the DISTAL trial (NCT05029414) and consisted of occlusions of the co- or nondominant branch of the M2 segment, M3 or M4 segment of the middle cerebral artery, A1, A2 or A3 segment of the anterior cerebral artery, P1, P2 or P3 segment of the posterior cerebral artery [7]. Further details on the classification of co- or nondominant M2 segments can be found in the Supplementary Appendix of the trial. In addition, cerebellar arteries were included in this study.

The primary outcome was defined as first-pass successful reperfusion (modified treatment in cerebral infarction (mTICI) ≥ 2b) in respect to the downstream territory of the occluded artery, and not regarding the whole territory of the M1 segment. The mTICI score was applied in the following manner:

“mTICI 0: no perfusion; mTICI 1: antegrade reperfusion past the initial occlusion, but limited distal branch filling with little or slow distal reperfusion; mTICI 2a (0–49%): antegrade reperfusion of less than half of the occluded target artery previously ischemic territory; mTICI 2b (50–89%): antegrade reperfusion of more than half of the previously occluded target artery ischemic territory; mTICI 2c (90–99%): near complete antegrade reperfusion of more than 90% of the previously occluded target artery ischemic territory; mTICI 3 (100%): complete antegrade reperfusion of the previously occluded target artery ischemic territory, with absence of visualized occlusion in all distal branches” [7]. Examples for mTICI scores in MDVO can be found in the Supplementary Information/Online Resource 1. In this study, successful reperfusion refers to a mTICI score of 2b or higher, near-complete reperfusion to a mTICI score of 2c or higher, and complete reperfusion to a mTICI score of 3.

Secondary outcomes including near-complete and complete reperfusion rates after the first and final pass, National Institutes of Health Stroke Scale (NIHSS) scores at 24 h and 7 days, as well as functional outcomes measured by the modified Rankin Scale (mRS) score at 7 and 90 days were assessed. The occurrence of symptomatic intracerebral hemorrhage (sICH) and procedural complications were collected as safety outcomes.

All data used for this study were collected during routine clinical care in accordance with the respective institution’s protocols. Hence, demographical data (age, sex, pre-stroke mRS) and data on the clinical condition (onset, NIHSS at admission) were retrieved by reviewing medical charts. Baseline non-invasive imaging data (imaging modality, Alberta Stroke Program Early CT Score (ASPECTS), CT-Perfusion, ischemic core volumes, penumbra volumes, location and side of vessel occlusion) were assessed by board-certified neuroradiologists, and angiographic images were assessed by the treating interventionalist. First-pass successful reperfusion, per-pass information on techniques, devices, and final reperfusion grades were evaluated. Furthermore, data on complications (vessel perforation, subarachnoid hemorrhage and emboli in new territories, intracranial hemorrhages according ECASS II, including sICH) and clinical outcomes (NIHSS at 24 h and 7 d, mRS at 7 d and 90 d, and mortality within 90d) were obtained.

Statistical analysis was performed by using Jamovi (version 2.3). [9] Patient characteristics were described by mean (standard deviation), median (interquartile range, IQR), or count (proportion) depending on the variable type and distribution. A sensitivity analysis to compare baseline characteristics of patients with available versus not available follow-up variables was performed.

## Results

A total of 85 cases were included from 11 sites, consisting of 47.1% (40/85) female and 52.9% (45/85) male patients, with a median age of 74.0 (IQR 67.0–81.0) years. Median NIHSS at admission was 9.0 (IQR 6.0–15.0). Baseline non-invasive imaging showed in 69.4% (59/85) an occlusion of co- or nondominant M2 segment, in 11.8% (10/85) of the M3 segment, in 8.2% (7/85) of the P2 segment, in 3.5% (3/85) of the A3 segment, and in 2.4% (2/85) of the A2 segment, P1 segment and superior cerebellar artery, respectively. Intravenous thrombolysis was administered in 32.9% (28/85), with a median onset-to-needle time of 119.0 min (IQR 91.8–188.8). Median ASPECT score was 10.0 (IQR 8.0–10.0). CT-Perfusion was performed in 84% (71/85) and showed median ischemic core volumes of 6.5 ml (IQR 0.0–18.0), and median salvageable tissue of 41.5 ml (28.8–68.3). Demographical, clinical and baseline non-invasive imaging data are shown in Table 1.

**Table 1:** Baseline Characteristics

	Overall (*n* = 85)
*Median age, years (IQR)*	74.0 (67.0–81.0)
*Female, n (%)*	40 (47.1)
*Median pre-stroke mRS*	0.0 (0.0–2.0)
*NIHSS score at admission (IQR)*	9.0 (6.0–15.0)
*Intracranial occlusion location, n (%)*	
Co- or nondominant M2	59 (69.4)
M3	10 (11.8)
A2	2 (2.4)
A3	3 (3.5)
P1	2 (2.4)
P2	7 (8.2)
Superior cerebellar artery	2 (2.4)
*Occlusion side, left, n (%)*	57 (67.1%)
*Median ASPECTS (IQR)*	10.0 (8.0–10.0)
*CT-Perfusion, yes, n (%)*	71 (84)
*Median ischemic core volume (CBF* *<* *30%), ml (IQR)*	6.5 (0.0–18.0)
*Median penumbra volume (Tmax* *>* *6* *sec), ml (IQR)*	41.5 (28.8–68.3)
*Intravenous thrombolysis, yes, n (%)*	28 (32.9%)
*Median onset-to-needle time, min (IQR)*	119.0 (91.8–188.8)

*ASPECTS* Alberta Stroke Program Early CT Score, *CBF* cerebral blood flow, *IQR* interquartile range, *mRS* modified Rankin Scale, *Tmax* Time-to-maximum

First-pass successful reperfusion (mTICI ≥ 2b) was achieved in 63.5% (54/85). First-pass near-complete (mTICI ≥ 2c) and complete reperfusion (mTICI 3) were achieved in 54.1% (46/85), and 43.5% (37/85), respectively. Final successful reperfusion was achieved in 96.5% (82/85), final near-complete reperfusion in 76.5% (65/85) and final complete reperfusion in 55.3% (47/85) of the cases. Overall median groin-to-reperfusion time was 21.5 min (IQR 13.0–33.3) and for EVTs terminated with only one pass, 17.0 min (IQR 10.0–28.0). Further EVT characteristics are found in Table 2.

**Table 2:** Endovascular Thrombectomy, overall and per first-line technique

	Overall (*n* = 85)	ADAPT (*n* = 36)	QUATTRO-ADAPT (*n* = 49)
*Onset-to-groin puncture*			
*N*-Miss	14	6	8
Median (IQR), min	255.0 (146.5–390.0)	221.5 (116.5–322.8)	273.0 (166.0–424.0)
*Groin-to-reperfusion*			
*N*-Miss	1	0	1
Median (IQR), min	21.5 (13.0–33.3)	20.5 (12.0–30.0)	23.0 (14.0–38.3)
*Number of passes, n (%)*			
1	53 (62.4)	22 (61.1)	31 (63.3)
2	18 (21.2)	10 (27.8)	8 (16.3)
3	9 (10.6)	4 (11.1)	5 (10.2)
4	4 (4.7)	0 (0)	4 (8.2)
5	1 (1.2)	0 (0)	1 (2.0)
*First-pass mTICI scores, n (%)*			
2b‑3 (Successful Reperfusion)	54 (63.5)	20 (55.6)	34 (69.4)
2c‑3 (Near-complete Reperfusion)	46 (54.1)	18 (50.0)	28 (57.1)
3 (Complete Reperfusion)	37 (43.5)	15 (41.7)	22 (44.9)
*Final mTICI scores, n (%)*			
2b‑3 (Successful Reperfusion)	82 (96.5)	34 (94.4)	48 (98.0)
2c‑3 (Near-complete Reperfusion)	65 (76.5)	26 (72.2)	39 (79.6)
3 (Complete Reperfusion)	47 (55.3)	20 (55.6)	27 (55.1)

*ADAPT* a direct aspiration first-pass technique, *IQR* interquartile range, *mTICI* modified treatment in cerebral infarction

**Fig. 1:**
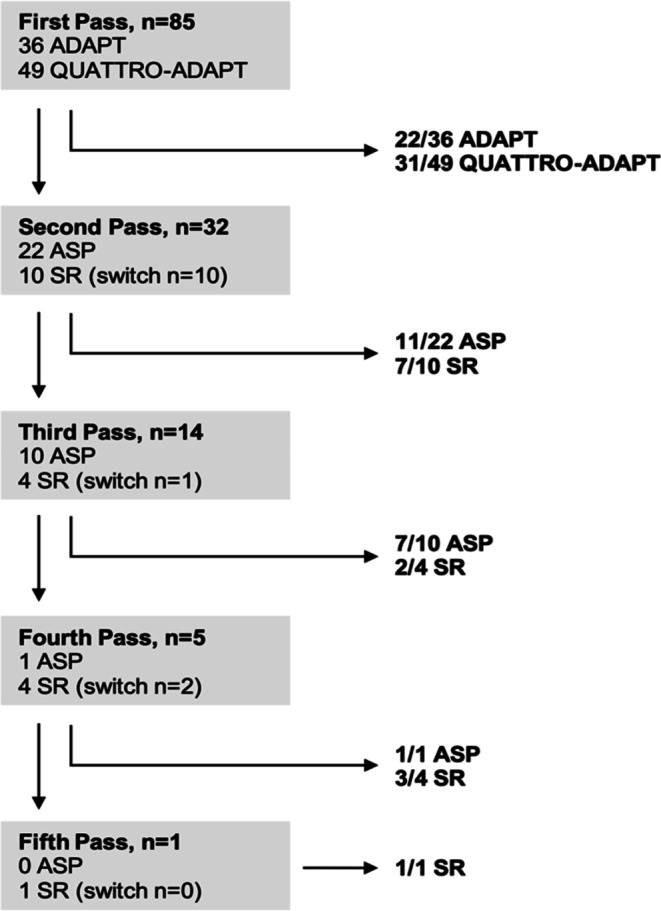
Overview of techniques, termination of EVT and timepoint of switching to the use of stent retrievers. ADAPT = a direct aspiration first-pass technique, ASP = aspiration, SR = stent retriever

A total of 53 interventions were terminated after the first pass, 18 after two passes, 9 after three, 4 after four, and 1 after five passes, as shown in Fig. 1. Rescue therapy with SR was deemed necessary in 15.3% (13/85) of the cases. Switching to SR happened in 10 of the 13 cases directly after the first pass, in 1 after the second pass, and in 2 after the third pass. Once having switched to the use of SRs, they continued to be used for all subsequent passes. In the cases that required more than one pass, and aspiration alone was continued throughout the whole intervention, successful reperfusion was achieved in 94.7% (18/19) of the cases.

**Fig. 2:**
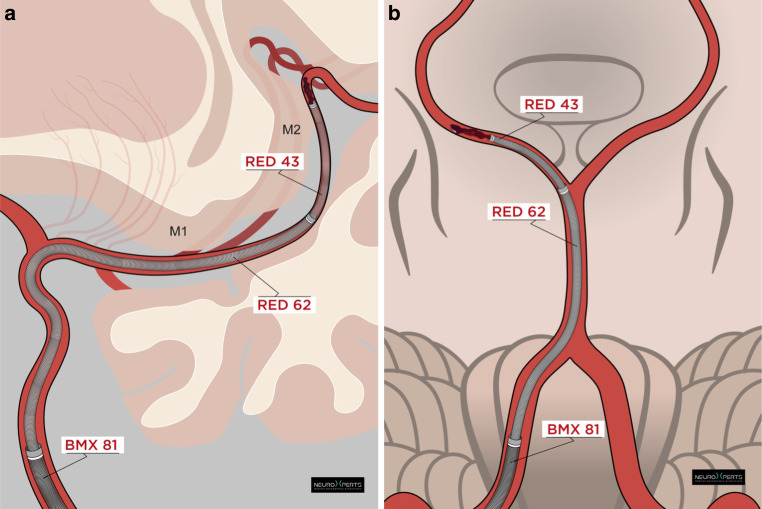
**a** QUATTRO-ADAPT for MDVO in the anterior circulation using a guide catheter (e.g., BMX81), a larger aspiration catheter (e.g., RED62), a smaller aspiration catheter (e.g., RED43), and a microcatheter and/or microwire (removed in this depiction for aspiration). The larger aspiration catheter is positioned in the distal M1 segment, acting as embolic protection device,** b** QUATTRO-ADAPT for MDVO in the posterior circulation using a guide catheter (e.g., BMX81), a larger aspiration catheter (e.g., RED62), a smaller aspiration catheter (e.g., RED43), and a microcatheter and/or microwire (removed in this depiction for aspiration). The larger aspiration catheter is positioned at the tip of the basilar artery, acting as embolic protection device

**Fig. 3:**
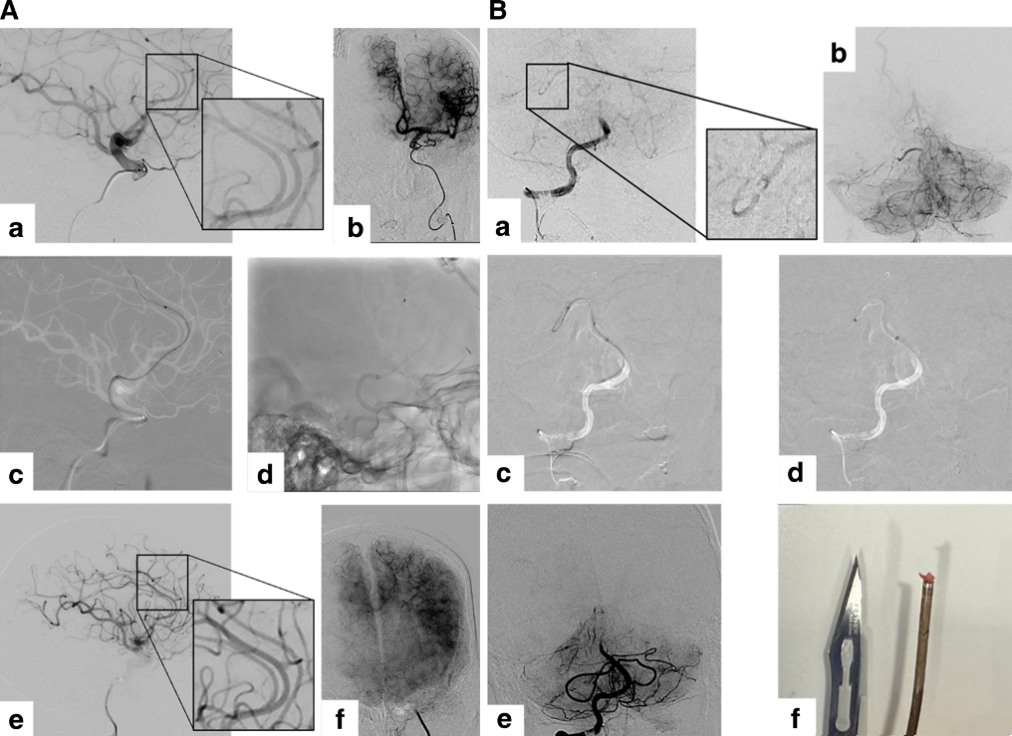
**a** 75 year old male patient with acute-onset, right-sided hemiparesis (NIHSS 15P) with an A3 occlusion on the left side (**a**, **b**). Using QUATTRO-ADAPT with a RED72 and RED43, microcatheter and microwire (**c**, **d**), complete reperfusion (mTICI 3) was achieved (**e**, **f**) with one pass and a groin-to-reperfusion time of 28 min. NIHSS score at discharge was 5P, **b** 63 year old male patient with acute-onset vertigo and emesis, gait instability, dysarthria and downbeat nystagmus with an occlusion of the superior cerebellar artery on the right side (**a**, **b**). Using QUATTRO-ADAPT with a RED62 and RED43, and microcatheter (**c**, **d**), complete reperfusion (mTICI 3) was achieved (**e**) with one pass and a white thrombus was removed (**f**). Apart from dysarthria, symptoms resolved within 24 h

QUATTRO-ADAPT was employed as first-line technique in 57.6% (49/85) of the cases. An illustration of the technique and case examples for the anterior and posterior circulation can be found in Figs. 2 and 3. First-pass successful reperfusion using QUATTRO-ADAPT was achieved in 69.4% (34/49). First-pass near-complete reperfusion was achieved in 57.1% (28/49), and first-pass complete reperfusion in 44.8% (22/49). Final successful reperfusion was achieved in 98.0% (48/49) in cases with QUATTRO-ADAPT as a first-line technique, final near-complete reperfusion in 79.6% (39/49) and final complete reperfusion in 55.1% (27/49). One vessel perforation (2.0%) was reported. Hemorrhagic transformation to parenchymal hematoma type 2 was not observed, and subarachnoid hemorrhage was detected in one case.

Of the total 85 cases, vessel perforation occurred in 2.4% (2/85), subarachnoid hemorrhage on postinterventional flat panel CT was observed in 4.8% (3/65), while flat panel CT was not performed in 20 cases. No emboli in new territories were reported, and access site hematoma was described in one patient. Intracranial hemorrhages according to ECASS II occurred in 18.8% (16/85) of the patients, whereof 9.4% are accounted as hemorrhagic infarction type 1, 2.4% as hemorrhagic infarction type 2, 4.7% as parenchymal hematoma type 1 and 2.4% (2/85) as parenchymal hematoma type 2. In three patients, subarachnoid hemorrhage was detected in the follow-up non-invasive imaging, and sICH was reported in one patient.

With a median NIHSS score of 3.5 at 24 h (IQR 1.8–10.3), we observed a median decrease of 4.5 NIHSS points (IQR 0.8–8.0) at 24 h and a median decrease of 5.5 NIHSS points (IQR 2.0–8.0) at 7 days, respectively. Median mRS score at 7 days was 3.0 (IQR 1.0–4.0), and at 90 days 2.0 (IQR 1.0–4.0). Functional independence (mRS 0–2) 90 days after the index stroke was regained in 58.7% (37/63), and excellent functional outcomes (mRS 0–1) were observed in 38.1% (24/63) of the patients. A median increase of 1.00 (IQR 0.0–3.0) from baseline mRS (0.0, IQR 0.0–2.0), at 7 days, as well as 90 days, was observed. In-hospital mortality during the initial hospitalization was reported in six cases, of which four had a known severe comorbidity; and in five patients after 90 days, of which two had a known severe comorbidity. An overview on clinical outcomes can be found in Table 3.

**Table 3:** Outcomes

	Overall (*n* = 85)
*Intracranial hemorrhage, n (%)*	
None	67 (78.8)
Hemorrhagic infarction type 1	8 (9.4)
Hemorrhagic infarction type 2	2 (2.4)
Parenchymal hematoma type 1	4 (4.7)
Parenchymal hematoma type 2	2 (2.4)
Subarachnoid hemorrhage	3 (3.5)
*sICH, yes, n (%)*	1 (1.2)
*Functional outcome at 90 days, n (%)*	
N‑Miss	22
Excellent functional outcome (mRS 0–1)	24 (38.1)
Functional independence (mRS 0–2)	37 (58.7)

*mRS* modified Rankin Scale, *sICH* symptomatic intracerebral hemorrhage

## Discussion

This retrospective study aimed to describe first-pass and final successful reperfusion rates of first-line aspiration with RED43 in patients with acute ischemic stroke due to primary, isolated MDVOs. Our analysis shows that first-line aspiration with RED43 can be effective and safe in in these patients using a triaxial, as well as quadriaxial approach (QUATTRO-ADAPT).

It has been reported that achieving near-complete reperfusion (mTICI ≥ 2c) with one pass results in significantly better functional outcomes compared to the same reperfusion grade achieved after more than one pass and has the potential to reduce procedural complications [10–12]. However, which technique is most effective as a first-line approach for MDVO treatment remains a subject of ongoing debate. An older meta-analysis based on observative data by Bilgin et al. suggested that a primary combined approach results in higher first-pass and final recanalization rates, fewer sICH and better functional outcomes in MDVOs compared to aspiration alone [13]. This is in line with a meta-analysis by Toh et al. which demonstrated higher odds for successful recanalization when SR are used in MDVO [14]. However, using SR in MDVOs has been associated with higher rates of subarachnoid hemorrhage and distal embolization compared to aspiration, [13] while EVT complications, such as vessel perforations and avulsions, have been reported to occur more frequently in MDVO thrombectomy compared to large vessel occlusion thrombectomy [15]. Furthermore, very low profile adaptable-mesh mechanical retrievers have shown high first-pass successful reperfusion rates (expanded TICI ≥ 2b67 of 70.5%) in very distal occlusions (A3, M3/4, P2/3 and superior cerebellar artery segments) but at the expense of 8.8% sICH complications [10–12]. Taken together, this implies that if more effective aspiration was achieved, this could realize the potential to combine high reperfusion rates (ideally with a single pass) with low procedural complications, ultimately resulting in better functional outcomes.

In our study, first-pass successful reperfusion was achieved in 63.5% of the cases. This finding surpasses previously described first-pass successful reperfusion rates of MDVO not only with ADAPT, but also of a primary combined approach (Bilgin et al. 52.4 and 54.9%, respectively).

First-pass near-complete reperfusion was observed in 54.1% of the cases in this study, which is higher to previously reported first-pass near-complete reperfusion rates with ADAPT (Toh et al. 48.3%). In the past, aspiration has repeatedly been thought to be inferior to a combined approach, with the reasoning that aspiration catheters were too large or too stiff for distal vessels [16]. The hypothesis that novel aspiration devices enable superior first-pass reperfusion rates is strengthened by the fact that the RED43 had not yet been introduced during the period of data collection of the studies included in the meta-analyses. In addition, a recently published retrospective study on primary and secondary MDVOs reported similar rates for near-complete first-pass reperfusion (57%) with the RED43 in primary MDVO [17].

Final successful reperfusion was achieved in 96.5% of the cases, which is substantially higher in comparison to final reperfusion rates of studies that did not include the RED43, be this randomized, prospective data or studies of observational nature (see Table 4). The same is applicable for final near-complete and final complete reperfusion. Although higher final reperfusion rates seem reasonable when using catheters specifically designed for distal vessels, an artificial augmentation of this value must be considered due to the retrospective nature of the data and the lack of a core-lab adjudicated reperfusion results. Additionally, the experience of the treating interventionalist, are factors that may influence the first-pass and final successful reperfusion rates. It has been shown that in case of a large vessel occlusion, the interventionalist’s experience and case volumes of EVTs per center are associated with successful reperfusion, functional outcomes [18] and in-hospital mortality [19, 20]. The participating centers in this study are mainly high-volume stroke centers, which further contributes to the understanding of the excellent reperfusion results.

**Table 4:** Final mTICI scores and safety outcomes with aspiration thrombectomy

	Bilgin et al. [13] (in %)	Toh et al. [14] (in %)	DISTAL, ASP group* (in %)	This study (RED43) (in %)
*Final mTICI score*				
2b–3 (Successful Reperfusion)	74.2	75.9	76.5	96.5
2c–3 (Near-complete Reperfusion)	Not reported	53.3	61.8	76.5
3 (Complete Reperfusion)	43.3	Not reported	38.2	55.3
*Safety Outcomes*				
Parenchymal hematoma type 2	Not reported	Not reported	5.6	2.4
Subarachnoid hemorrhage	1.8	Not reported	19.4	3.5
sICH	8.5	9.2	2.8	1.2

*manuscript in preparation

*ASP* aspiration, *mTICI* modified treatment in cerebral infarction, *sICH* symptomatic intracerebral hemorrhage

QUATTRO-ADAPT was used in 49 cases as a first-line technique and resulted in 69.4% of the cases in successful first-pass reperfusion. Notably, for almost all reperfusion groups (successful, near-complete and complete reperfusion), QUATTRO-ADAPT had higher first-pass and final reperfusion rates than simple ADAPT. For instance, first-pass successful reperfusion was observed in 69.4% with QUATTRO-ADAPT, and 55.6% with ADAPT. The incidence of procedural complications, hemorrhagic transformations and symptomatic intracerebral hemorrhages are overall low, making a comparison between the triaxial and quadriaxial technique negligible. QUATTRO-ADAPT seems safe and efficient, with potential to surpass triaxial approaches.

### The QUATTRO-ADAPT technique

The QUATTRO-ADAPT technique features four catheters: a guide catheter, a larger aspiration catheter (e.g., a RED62) a smaller aspiration catheter (e.g., a RED43), and a microcatheter plus microwire. After accessing the arterial vasculature and positioning the guide catheter in the internal carotid or vertebral artery, the larger aspiration catheter is advanced to the distal M1 segment of the middle cerebral artery, the carotid T (for anterior cerebral artery occlusions), or the basilar artery (for posterior circulation occlusions). The smaller aspiration catheter is then advanced over the microcatheter/microwire as close to the thrombus as possible. The microcatheter/microwire are then removed, which increases the aspiration lumen in the RED43 [21]. Aspiration through the smaller catheter is initiated using an aspiration pump after reaching the face of the clot. To maintain the negative pressure, the pump is exchanged with a 60 ml Luer lock syringe, and the pump subsequently connected to the larger aspiration catheter. Aspiration at the larger aspiration catheter is only initiated as soon as the smaller aspiration catheter and clot are retrieved at the tip of the large aspiration catheter. This can mitigate the risk of vessel collapse [22]. Ultimately, the smaller aspiration catheter is removed from the patient, while the larger aspiration catheter remains in its place (distal M1, carotid T, or basilar artery).

The idea behind the technique—having a large aspiration catheter in the vessel prior to the occluded branch—is coming from large vessel occlusion thrombectomy. As balloon-guide catheters are used in the internal carotid artery or large sheaths in the distal internal carotid artery for embolic protection when performing M1 thrombectomy, the same can be done with a large aspiration catheter placed in the M1, when performing M2/M3 reperfusion. As clots are not always ingested in the RED43, the inclusion of a large proximal aspiration catheter acts as an embolic protection device. With additional proximal aspiration, potential emboli in new territories or distal emboli can be minimized, which is supported by the higher rates of excellent reperfusion with the QUATTRO-ADAPT technique. This is especially important in the posterior circulation, as the usual approach is to have a guide catheter in the distal vertebral arteries and retrieve the RED43 all the way from the P2 segments to the guide catheter in the vertebral artery, with potentially a clot at the tip. The obvious danger is to lose micro-/or macro-emboli in the basilar branches, with severe clinical consequences (see Supplementary Information/Online Resource 1). Adding a larger aspiration catheter (e.g., a RED62) in the distal basilar, negates this risk. Additionally, when performing MDVO aspiration thrombectomy, it is crucial to depict the face of the clot for exact placement of the catheter tip. The inclusion of a large aspiration catheter in the M1 or distal basilar makes this possible, as opposed to an angiography series from the proximal carotid. Furthermore, if there is the need for a second pass, having an aspiration catheter in the M1 or distal basilar provides an excellent opportunity for fast navigation to the occluded segment.

Overall, we observed low rates of procedural complications, such as vessel perforations (*n* = 2), emboli in new territories (*n* = 0) or subarachnoid hemorrhages (*n* = 3, 3.5%). Both vessel perforations did not result in sICH, and the two patients regained functional independence. Hemorrhagic transformations were barely detected on follow-up imaging, with parenchymal hematoma type 2 in two (2.4%), and sICH in one patient (1.2%). Comparing the prevalence of sICH to randomized, prospective data and data on earlier aspiration devices, the rate reported in this study shows a trend towards a lower incidence of sICH.

Despite higher rates of successful reperfusion, functional outcomes were comparable to randomized, prospective data; with a median mRS at 90 days of 2.0, 58.7% of patients regaining functional independence (DISTAL 55.2%, ESCAPE-MeVO 54.1%) and 38.1% reaching excellent functional outcome (DISTAL 34.7%, ESCAPE-MeVO 41.6%). This could be explained by higher NIHSS scores at admission in this study with a median of 9.0 (IQR 6.0–15.0), compared to DISTAL (median 6.0, IQR 5.0–9.0) and ESCAPE-MeVO (median 8.0, IQR 5.0–11.0). However, functional outcomes at 90 days should be interpreted carefully in this retrospective analysis, as 22 values are missing. Patients without available mRS at 90 days had a higher median pre-stroke mRS compared to the group of patients with available mRS at 90 days (2.0, IQR 0.0–2.0 versus 0.0, IQR 0.0–1.0, *p* = 0.003). While no other difference in baseline characteristics between these two groups was detected, this implies that functional outcomes may be overestimated due to attrition of patients with higher pre-stroke mRS.

The main limitation of this study is its retrospective nature. As we included only data collected during clinical routine, some variables—especially follow-up variables—have not always been collected. Furthermore, e.g. thrombus composition is not collected in clinical routine, but may influence reperfusion results. All cases that had been performed at the centers with first-line aspiration using RED43s were included, while the main reason for exclusion were the secondary nature of the occlusion or a primary combined approach as a first-line technique; both pre-specified exclusion criteria. In the majority of cases, the occlusion was located in the co- and non-dominant M2 segment. Consequently, the findings should be extrapolated with caution to more distal vessel occlusions. Lastly, our patient collective remains restricted to European and North American ethnicities.

This retrospective study shows that first-line aspiration with the RED43 in patients with acute ischemic stroke due to MDVOs can be safe and effective. The same applies for the quadriaxial approach, QUATTRO-ADAPT. Larger, prospective data sets with novel aspiration devices, such as the RED43, are needed to determine whether first-line aspiration is the optimal technique for thrombectomy in MDVO patients.

## Supplementary Information

The supplementary information includes an overview of mTICI scoring in medium or distal vessel occlusions as applied in this study and further elaboration on a potential mechanism of emboli to new territories using a triaxial aspiration technique.

## Data Availability

The data that support the findings of this study are available from the corresponding author upon reasonable request.

## Abbreviations

ADAPT: A Direct Aspiration First Pass Technique
ASPECTS: Alberta Stroke Program Early CT Score
DISTAL: EnDovascular therapy plus best medical treatment (BMT) versus BMT alone for MedIum VeSsel Occlusion sTroke—a prAgmatic, international, multicentre, randomized triaL
ECASS: European Cooperative Acute Stroke Study
ESCAPE-MeVO: EndovaSCular TreAtment to imProve outcomEs for Medium Vessel Occlusions trial
EVT: Endovascular thrombectomy
MDVO: Medium or distal vessel occlusion
mRS: Modified Rankin Scale
mTICI: Modified Treatment In Cerebral Infarction
NIHSS: National Institutes of Health Stroke Scale
RED43: RED43 Reperfusion Catheter
sICH: Symptomatic intracerebral hemorrhage
SR: Stent retriever
