# Supplementary material for: First-line Aspiration Thrombectomy with the RED43 Catheter for Acute Ischemic Stroke due to Medium or Distal Vessel Occlusion
Source: Clin Neuroradiol. 2025 Sep 29;36(1):185–94. doi: 10.1007/s00062-025-01567-8 (PMC13008998; doi:10.1007/s00062-025-01567-8)
Supplement: Supplementary file 1 — The supplementary information includes an overview of mTICI scoring in medium or distal vessel occlusions as applied in this study and further elaboration on a potential mechanism of emboli to new territories using a triaxial aspiration technique. [file 62_2025_1567_MOESM1_ESM.pdf]

Supplementary Information to:

First-line Aspiration Thrombectomy with the RED43 Catheter for Acute Ischemic Stroke due to Medium or Distal Vessel Occlusion, *Clinical Neuroradiology*

Vera Aebischer, Alex Brehm, Nikki Rommers, Alejandro Spiotta, Mohammad Sowlat, Karthik Raghuram, Justin M Cappuzzo, Jeffrey Beecher, Charles Matouk, Abdelaziz Amllay, Axel Rohr, Manraj Heran, David Volders, Pascal J Mosimann, Mohammad Al-Tibi, Johan Wassélius, Björn M Hansen, Markus Holtmannspoetter, Martin Schloesser, Stephan Meckel, Oumaima Aoua, Suresh Giragani, Andrea Boghi, Nikolaos Ntoulias, Victor Schulze-Zachau, Ioannis Tsogkas, Kristine A Blackham, Aikaterini Anastasiou, Marios Psychogios

Corresponding author: Vera Aebischer, Department of Diagnostic and Interventional Neuroradiology, University Hospital Basel and University of Basel, Petersgraben 4, 4031 Basel, Switzerland, vera.aebischer@usb.ch

mTICI scores in medium or distal vessel occlusions

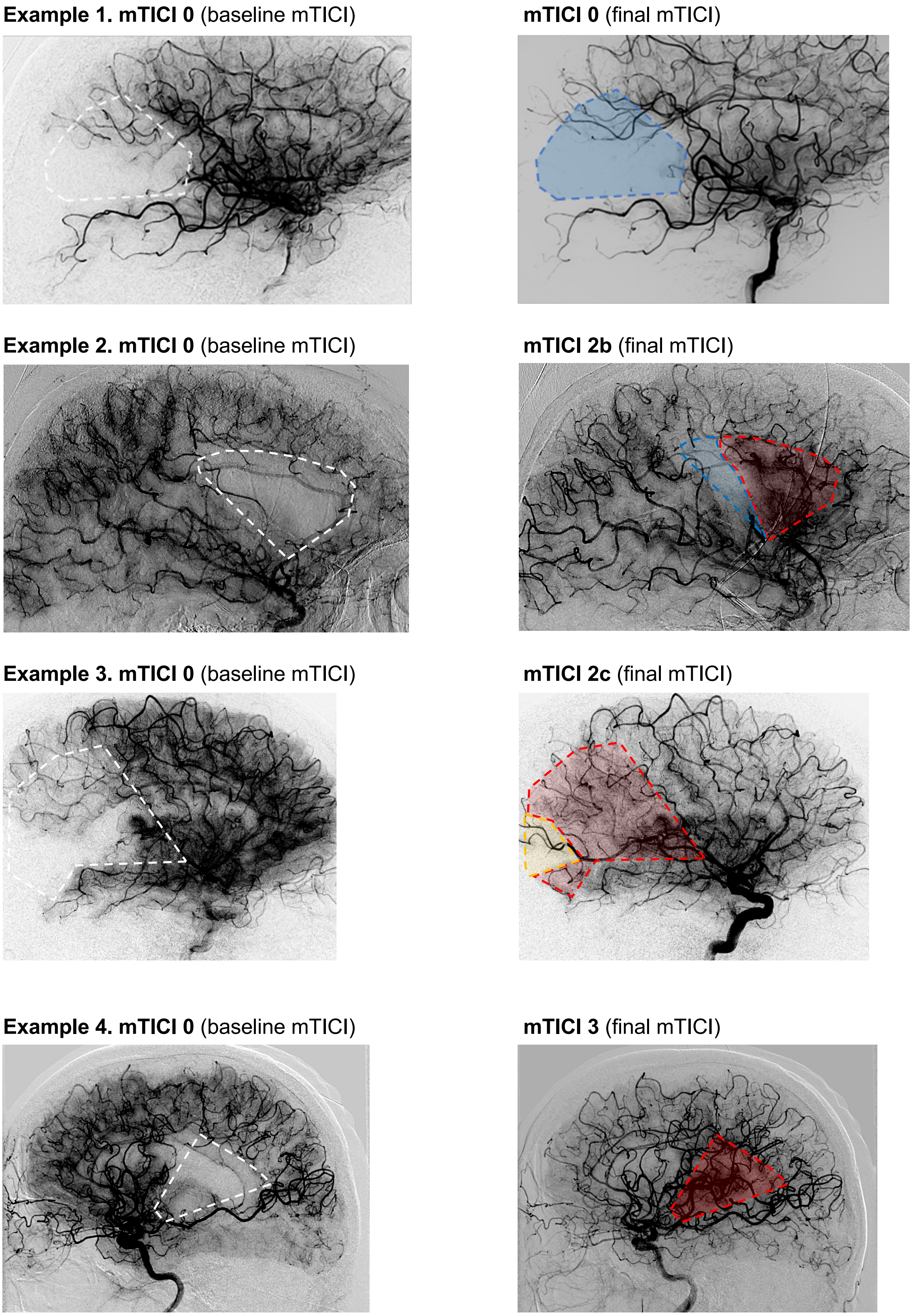

Online Resource 1A mTICI scores in MDVO. Baseline perfusion deficits in territories downstream to the occlusion are marked in white. Reperfused territories are marked in red, territories with slow flow in yellow, and territories without reperfusion in blue.

Potential emboli in a triaxial approach

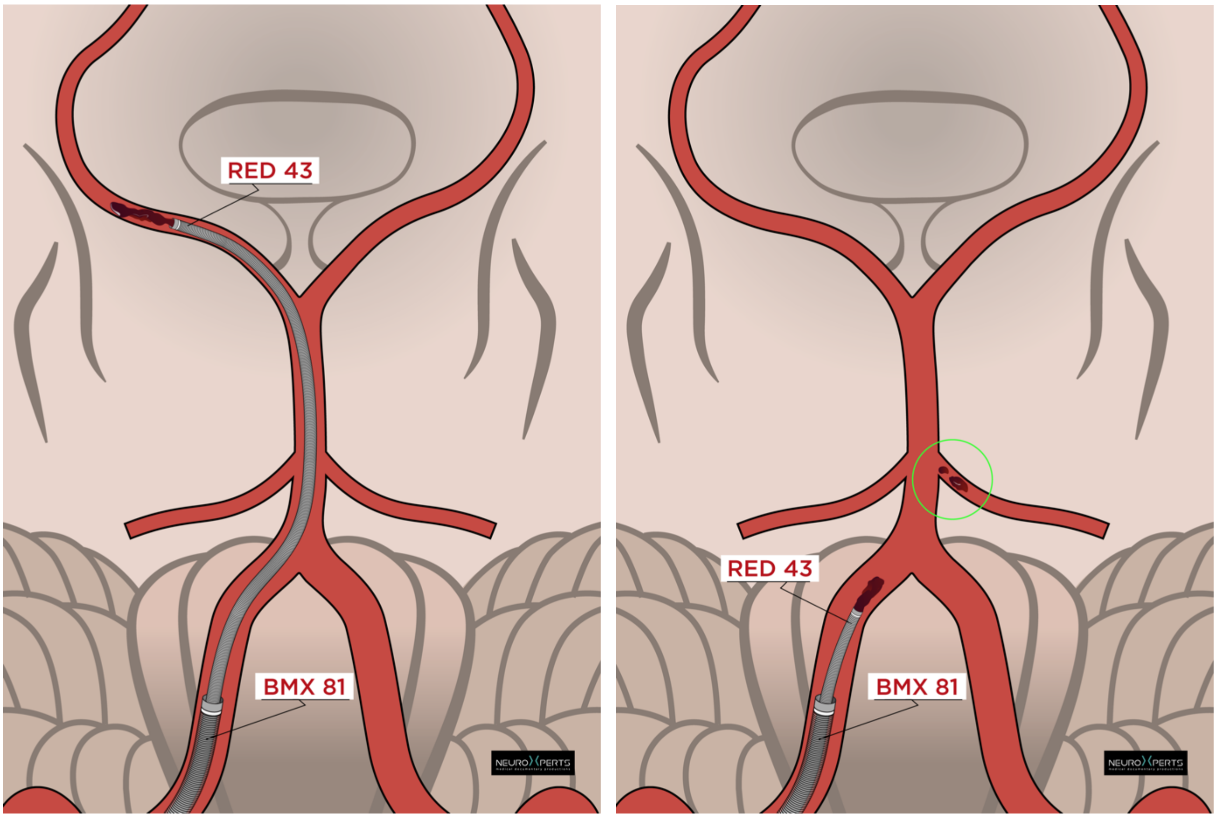

Online Resource 1B A direct aspiration first-line technique (triaxial). The danger is to lose micro-/or macro-emboli in the basilar branches, with severe clinical consequences. Adding a large aspiration catheter, e.g. the RED62, in the distal basilar, negates this risk (see manuscript Fig. 2)
